# Microbial Contamination of Drinking Water and Human Health from Community Water Systems

**DOI:** 10.1007/s40572-014-0037-5

**Published:** 2015-01-27

**Authors:** Nicholas J. Ashbolt

**Affiliations:** School of Public Health, University of Alberta, Edmonton, Room 3-57D, South Academic Building, Alberta, T6G 2G7 Canada

**Keywords:** Water safety plans, HACCP, QMRA, Surrogate, Indicator, Antimicrobial resistance, Enteric pathogen, Opportunistic pathogen, Viruses, Bacteria, Parasitic protozoa, Fungi, Waterborne, Water-based, Risk management

## Abstract

A relatively short list of reference viral, bacterial and protozoan pathogens appears adequate to assess microbial risks and inform a system-based management of drinking waters. Nonetheless, there are data gaps, e.g. human enteric viruses resulting in endemic infection levels if poorly performing disinfection and/or distribution systems are used, and the risks from fungi. Where disinfection is the only treatment and/or filtration is poor, cryptosporidiosis is the most likely enteric disease to be identified during waterborne outbreaks, but generally non-human-infectious genotypes are present in the absence of human or calf fecal contamination. Enteric bacteria may dominate risks during major fecal contamination events that are ineffectively managed. Reliance on culture-based methods exaggerates treatment efficacy and reduces our ability to identify pathogens/indicators; however, next-generation sequencing and polymerase chain reaction approaches are on the cusp of changing that. Overall, water-based *Legionella* and non-tuberculous mycobacteria probably dominate health burden at exposure points following the various societal uses of drinking water.

## Introduction

The provision of safe drinking water has been one of humanity’s most successful public health interventions and is a defining aspect of a developed country. Nonetheless, ignorance of the potential risks and inappropriate training of staff and managers working on drinking water systems still results in unnecessary waterborne disease outbreaks in affluent communities [1•]. Furthermore, re-introduction of once-controlled diseases, such as cholera, may rapidly spread during periods of disasters when sanitation systems are non-functional and drinking water treatment is inadequate. A recent example was the Haitian epidemic [2] where, although a developing region, international aid workers introduced the outbreak strain and then tourists spread infections to more developed regions [3, 4•]. Hence, to some degree, differentiating pathogen risks between developed regions and those less developed, particularly rapidly developing regions, is artificial and not very useful. Therefore, this review is relevant to most regions with functional drinking water treatment provided through a community system. A key realization is the need for ongoing system-wide vigilance, coupled with a preventative rather than just responsive management approach. This approach is best practiced globally using principles from the food industry’s Hazard Analysis Critical Control Point (HACCP) approach, described by the World Health Organization (WHO) as Water Safety Plans (WSPs) [5, 6]. In addition to existing regulatory framework constraints, this WSP approach may be particularly hard to implement in (developed) regions that have not identified major waterborne outbreaks for decades.

Even with well-operated drinking-water treatment systems, there is growing concern that aging drinking water distribution systems (DWDSs) are vulnerable to higher rates of mains breaks/repairs and related pressure losses that may lead to pathogen intrusion scenarios [7•, 8•]. Also, traditional end-of-pipe compliance monitoring practices may not identify short periods of DWDS intrusions or short periods of poorer treatment performance, such as is associated with rain-induced dirty-water events that appear to be associated with increased rates of waterborne gastrointestinal disease [9]. Generally, drinking-water gastrointestinal cases are not well quantified, even in developed regions, due to the insensitivities of surveillance and specific epidemiology studies [10, 11]. For the US (with approximately 300 million people) estimates of annual drinking-water gastrointestinal cases range from 12–19 million [12]. In addition, beyond the DWDS is a vast network of building or in-premise plumbing that, under certain conditions, allows the growth and release of water-based opportunistic pathogens, many resulting in respiratory or skin diseases, such as from *Legionella pneumophila* and non-tuberculous mycobacteria (NTM) [13]. At least in the US, these water-based pathogens appear to cause a higher health burden via hospitalization than waterborne enteric pathogens [14•]. With the exception of recently enacted regulatory monitoring for *Legionella* in The Netherlands, France, and Germany, these water-based pathogens are neither targeted nor identified by current regulatory monitoring that focuses on fecal indicator bacteria (FIB) [e.g. *Escherichia coli* and enterococci]. Due to environmental growth of water-based opportunistic pathogens, quite different but familiar control strategies (elimination of stagnation zones and related temperature and disinfectant control) are required for in-premise plumbing, particularly in healthcare settings [15].

This review builds on previously conducted reviews [5, 16•, 17–19], and is organized around recent findings associated with drinking-water microbial hazards and scenarios that need to be managed as part of a WSP-like system-wide management framework to provide safe drinking water. This information should also be informative for the growing use of quantitative microbial risk assessment (QMRA) to inform WSPs [20]. As such, pathogens are grouped into waterborne (enteric viruses, bacteria, parasitic protozoa, and fungi) and water-based (environmental viruses, bacteria, free-living protozoa, and fungi) groups. Furthermore, crosscutting pathogen issues, such as antimicrobial resistance (AMR) gene transfer and the role of the host microbiome, are introduced.

## Waterborne (Enteric) Pathogens

There are over 500 waterborne pathogens of potential concern in drinking waters, identified by the US Environmental Protection Agency (EPA) through its Candidate Contaminant List (‘CCL 3 Universe’ list, available at http://www.epa.gov/safewater/ccl/pdfs/report_ccl3_microbes_universe.pdf). To aid in identifying representatives within each of the microbial groups (viruses, bacteria, parasitic protozoa, and fungi), Table 1 lists current and likely members of importance to manage waterborne risks from community drinking waters. This subset contains representative members, known as reference pathogens, which are increasingly being used to support WSPs via QMRA, given their general coverage of the vast majority of human health effects associated with pathogens in drinking water [26].

**Table 1:** Recognized and potential enteric and water-based microbial pathogens to manage community drinking water risks

Microbial group	Enteric (waterborne)		Water-based (opportunistic)	
	Recognized	Potential	Recognized	Potential
Viral	*Adenovirus* 40 and 41 *Avipolyomavirus* ^a^ *Enterovirus* A–D *Hepatitis* A and E *Norovirus* G1 and G2 *Rotavirus* A *Sapovirus* G1	*Mamastrovirus* 1 *Orthoreovirus* C	None	*Mimivirus* ^b^ *Mamavirus* ^b^
Bacterial	*Aeromonas hydrophila* ^c^ *Campylobacter coli* *C. jejuni* *Salmonella enterica* (non-typhoid) *Shigella sonnei* *Vibrio cholerae* ^e^	*Acinetobacter baumannii* ^c^ *Arcobacter butzleri* *Helicobacter pylori* *Clostridium difficile* ^c^ *Listeria monocytogenes* *Pseudomonas aeruginosa* ^c^ *Staphylococcus aureus* ^c^ *Yersinia enterocolitica*	*Legionella longbeacheae* ^d^ *Legionella micdadei* ^d^ *Legionella pneumophila* ^d^ *Escherichia coli* O157:H7^f^ NTM^c,d,g^ *Pseudomonas aeruginosa* ^c^	*Acinetobacter baumannii* ^c^ *Aeromonas hydrophila* ^c^ ARB (*Afipia, Bosea, Parachlamydia* spp., *Coxiella burnetii*)^d^ *E. coli* (toxigenic strains) *Listeria monocytogenes* ^d^ *Staphylococcus aureus* ^c^ *Stenotrophomonas maltophilia* ^c,d^
Protozoan	*Cryptosporidium hominis* and *parvum* *Cyclospora cayetanensis* *Giardia intestinalis* assemblages A and B *Toxoplasma gondii*	*Blastocystis hominis*	*Acanthamoeba T4* *Balamuthia mandrillaris* *Naegleria fowleri*	*Acanthamoeba* spp*.* ^d^ *Vahlkampfia* spp.^d^ *Vannella* spp*.* ^d^ *Vermamoeba vermiformis* ^d^
Fungal	None Microsporidia (e.g. *Encephalitozoon bieneusi, E. intestinalis*)	*Candida albicans*	None	*Aspergillus fumigatus* *Aspergillus terreus* *Candida albicans* *Candida parapsilosis* *Exophiala dermatitidis*

*AMR* antimicrobial-resisting, *ARB* amoeba-resisting bacteria, *QMRA* quantitative microbial risk assessment, *VBNC* viable but non-culturable, *DWDS* drinking water distribution systems, *NTM* non-tuberculous mycobacteria

^a^Main species being *JC polyomavirus,* which is largely excreted in urine, as it infects the kidneys along with the respiratory system or brain

^b^ *Acanthamoeba polyphaga mimivirus* (APMV) may cause respiratory disease and unknown health effects from *Mamavirus* [21]

^c^Most strains of species from the environment may be non-pathogenic, however there is future potential for AMR strains. For *P. aeruginosa*, most clinical disease is identified with *otitis media*, with less severe disease via drinking water aerosols leading to diffuse bronchopneumonia and more severe disease in high-risk children with cystic fibrosis; folliculitis is important directly via drinking waters used in pools/spas

^d^Largely non-pathogenic amoeba hosts containing ARB, of which many ARB are of key concern, from drinking water [22], except possibly *Coxiella burnetii*, which is of low theoretical risk estimated by QMRA [23•]

^e^Cholera may re-emerge if a major event interferes with drinking-water disinfection, and an epidemic strain is introduced from an endemic region of the world

^f^Shiga toxin and verotoxin-producing *E. coli* (and various intracellular *Salmonella* and *Listeria*) strains may grow within free-living protozoa [24], and non-pathogenic VBNC *E. coli* in DWDS biofilms [25]

^g^Various NTM, including *Mycobacterium avium* (*M. intracellulare*) complex, *M. chelonae, M. fortuitum, M. gordonae,* and *M. kansasii*

Various counties have developed treatment goals or drinking-water parameters based on microbial risk assessment [5]; however, only in the Netherlands is there a regulatory requirement for drinking-water companies to provide water that, in theory, meets an annual gastrointestinal risk of <10^−4^ 95 % of the time. This means, for example, that drinking water is required to have less than one enteric virus per million liters of drinking water [27], a concentration well below the capabilities of current measurement techniques. Epidemiology studies have shown an increased gastrointestinal risk (30 %) when enteric virus concentrations were at approximately one genomic copy per liter [28], highlighting the difference between what can be measured directly compared with the low concentrations sought by regulations to control risk of gastrointestinal illness as estimated by QMRA. Hence, estimations of enteric pathogen risks are more reliant upon measuring pathogen concentrations in contaminated source water(s), and using surrogates to estimate treatment removals [29]. As such, QMRA estimates contain uncertainties associated with reference pathogen detection, their relationships to surrogates used, and relevance of limited dose–response models that may not address life-stage of most interest. Therefore, QMRA is probably better used to present relative risks to inform management about different risk scenarios in their considerations for developing WSPs, rather than trying to estimate absolute risk levels.

Key areas not currently addressed in most QMRAs of systems are the DWDS and premise plumbing risks. For both, biofilms on pipe walls and sediments within storage reservoirs and pipes [30] present a sequestering environment for various enteric pathogens, including viruses that are relatively resistant to normal disinfection treatments [31], and allow growth of water-based pathogens [32]. Limited QMRA studies of biofilm enteric pathogen risks are available but results suggest that accumulation of virions, known to occur within DWDSs [28], could present a higher level of risk than background levels when they slough off and re-enter the mass flow to customers [33]. In addition, various free-living protozoa and metazoan feeding within DWDS biofilms may also act as disinfectant-resistance transport hosts for enteric pathogens, including viruses [34•, 35]. The risks associated with water-based pathogens, of particular relevance to premise plumbing, are discussed latter.

### Reference Enteric Viruses

Human noroviruses cause the most gastrointestinal illness in all regions of the world, with the vast majority thought to be acquired via person-to-person and then by food [36•], given the predominance of genogroup II strains implicated. In waterborne cases, genogroup I is normally implicated [37], presumably due to increased environmental robustness. An interesting finding with human noroviruses and the second most common cause of gastrointestinal illness, rotavirus (although greatly diminishing due to childhood vaccination programs), is the need for certain histo-blood group antigen (HBGA) receptors for these pathogens to bind to target cells [38]. Not only do certain gut bacteria have these HBGA binding sites but these bacteria may also facilitate infection, as recently demonstrated with human B cells [39•]. Therefore, one’s gut microbiome and blood group impact the likelihood of infection. Furthermore, there is now optimism that a routine cell culture system for human noroviruses may be developed, which would be of particular value to the water-treatment industry. Non-human, culturable noroviruses, such as murine noroviruses among others, are used as surrogates for treatment performance (inactivation studies) but there is limited understanding of the validity of these surrogates for any human norovirus genogroup or mode of inactivation [40, 41•].

Overall, *Norovirus* is considered one of the most conservative virus targets for drinking-water QMRA studies (measured via reverse transcriptase-quantitative polymerase chain reaction [RT-qPCR])—conservative in that if *Norovirus* risks are managed, most other enteric viruses will also be controlled. However, there are limited dose–response models to interpret molecular (RT-qPCR) exposure data [42•], noting it is unknown what fraction of virions may be infectious (given that fresh suspensions of virions were used in dose–response studies versus more aged virions typically present in the environment, and that both infectious and non-infectious virions are measured by RT-qPCR). Counteracting the infectious fraction to some unknown degree is virion clumping, which would increase infection likelihood [43]. In the absence of infectivity data, the WHO, as well as various other jurisdictions, recommend culturable rotaviruses as a preferred enteric virus reference, or the environmentally hardier human adenoviruses or reoviruses, which may be in similarly high concentrations as noroviruses in sewage-contaminated waters [44–46]. The problem with using adenoviruses is that gastrointestinal illness is largely caused by serogroups 40 and 41, whereas the dose–response model available for QMRA is only for a respiratory strain, and there are limited illness or infection rates known for reoviruses (*Orthoreovirus C*) in humans.

Regarding virus surrogates (typically bacteriophages), there is no identified single surrogate known to mimic the various behaviors of different human enteric viruses (with respect to surface charge, hydrophobic interactions, and inactivation by sunlight, disinfectants, etc.). Human adenoviruses provide a good example of a key virus risk and considerations for what surrogate is most suited, being more resistant than *Enterovirus* or *Rotavirus* when ultraviolet (UV) disinfection is practiced. Given the expectation for a 4-log_10_ inactivation of surface waters in the US (i.e. 99.99 %), it is of concern that human adenoviruses are relatively poorly inactivated by so-called low pressure (LP) UV disinfection (monochromatic at 254 nm), but readily inactivated with medium pressure (MP), polychromatic UV treatment (including 254, 265, 280 and 295 nm wavelengths) [47•]. The differences are, in part, thought to arise due to LP-UV targeting nucleic acids, which may be repaired by the host cell, whereas MP-UV also impairs the protein coats of virions, potentially impeding cell infection.

Therefore, consideration of these different modes of inactivation is important when selecting an appropriate surrogate. For example, a commonly used disinfection bacteriophage surrogate, the F-RNA coliphage MS2, demonstrates a 4-log inactivation with 64 mJ/cm^2^ LP-UV and 46 mJ/cm^2^ MP-UV compared with adenovirus 2 requiring 120 and 45 mJ/cm^2^, respectively [47•]. Not only do these LP- and MP-UV systems inactivate bacteriophages by different mechanisms but their host bacterial cells may also use different repair mechanisms (referred to as photoreactivation and dark repair mechanisms). In the case of MS2, there appears to be no repair by the *E. coli* host, whereas the dsDNA *Salmonella* bacteriophage PRD1, appears to be the most resistant of four bacteriophages examined, with a 4-log_10_ reduction (similar for both LP- and MP-UV) requiring 103 mJ/cm^2^ with photoreactivation versus only 35 mJ/cm^2^ without reactivation [47•]. In general, MS2 is a valid surrogate for chemical disinfection processes but due to its *E. coli* source from various warm-blooded animals, it is not a good index of human enteric virus presence in environmental waters [48].

In summary, human enteric reference viruses include species of the genera *Adenovirus, Enterovirus, Norovirus, and Rotavirus,* and potentially *Orthoreovirus C* (a reovirus). Generally, only one or two are chosen but only when there is likely to be a human source of fecal contamination yielding human enteric virus risk. In the absence of a sanitary survey to indicate possible sewage/septic seepage pollution to source waters, or to give a second opinion, human-targeted *Bacteroides* provide a valuable and potentially more sensitive indicator than assaying for human enteric viruses [49]. In urbanized settings, DWDS intrusions are likely to include human enteric viruses, which may dominate gastrointestinal risks via drinking water [43].

### Reference Enteric Bacteria

The classic waterborne enteric pathogens include *Vibrio cholerae* (serogroups O1 and O139, causing cholera)*, Salmonella enterica* (subsp. *enterica* ser. Typhi, causing typhoid), and *Shigella* spp. (four species causing dysentery), which have largely been controlled by water treatment/disinfection and are therefore rarely an issue via drinking water in developed regions. However, person-to-person and foodborne spread maintains *Shigella sonnei* within the sewage of developed regions, along with closely-related shiga toxin and verotoxin-producing *E. coli,* and pathogenic species of *Campylobacter, Salmonella, Arcobacter, Helicobacter and Yersinia* (Table 1). An emerging issue is that of AMR, which may occur within any of the bacterial members listed in Table 1 but is noted here by example for *E. coli* in well waters associated with animal production [50]. These AMR genes may horizontally transfer between commensal and enteric pathogenic bacteria, and present a higher risk due to antimicrobial treatment failures [51•]. Within healthcare facilities, there is also a considerable health burden due to the prevalence of AMR *Pseudomonas aeruginosa* and *Clostridium difficile*; with the latter being a spore-former it may persist in sewage and river waters and eventually make its way to drinking waters, and AMR-*P. aeruginosa* may grow post-water treatment (discussed further in the section on “Control of Water-Based Pathogens”) [Table 1, potential future concern]. AMR *Staphylococcus aureus* is also of potential concern via companion animals to water [52] and could be considered a useful reference pathogen for AMR in the future.

The most recognized and useful reference enteric bacteria in developed regions are *Salmonella enterica, Campylobacter jejuni*, and *E. coli* O157:H7, each containing human pathogenic strains that vary by fecal source [53, 54]. However, there is limited data on the quantification of these pathogens in source drinking waters (most studies provide presence/absence data) due to the difficulties in culturing these bacteria from the environment [31, 55]. Therefore, given the general presence of FIB and their ease of culture, either *E. coli* or enterococci have been used as surrogates to assess enteric bacterial pathogen removals by treatment barriers. However, there are improvements in risk management if culture-free methods are used, as discussed in the next section.

### Non-Culturable States of Enteric Bacteria

**Fig. 1:**
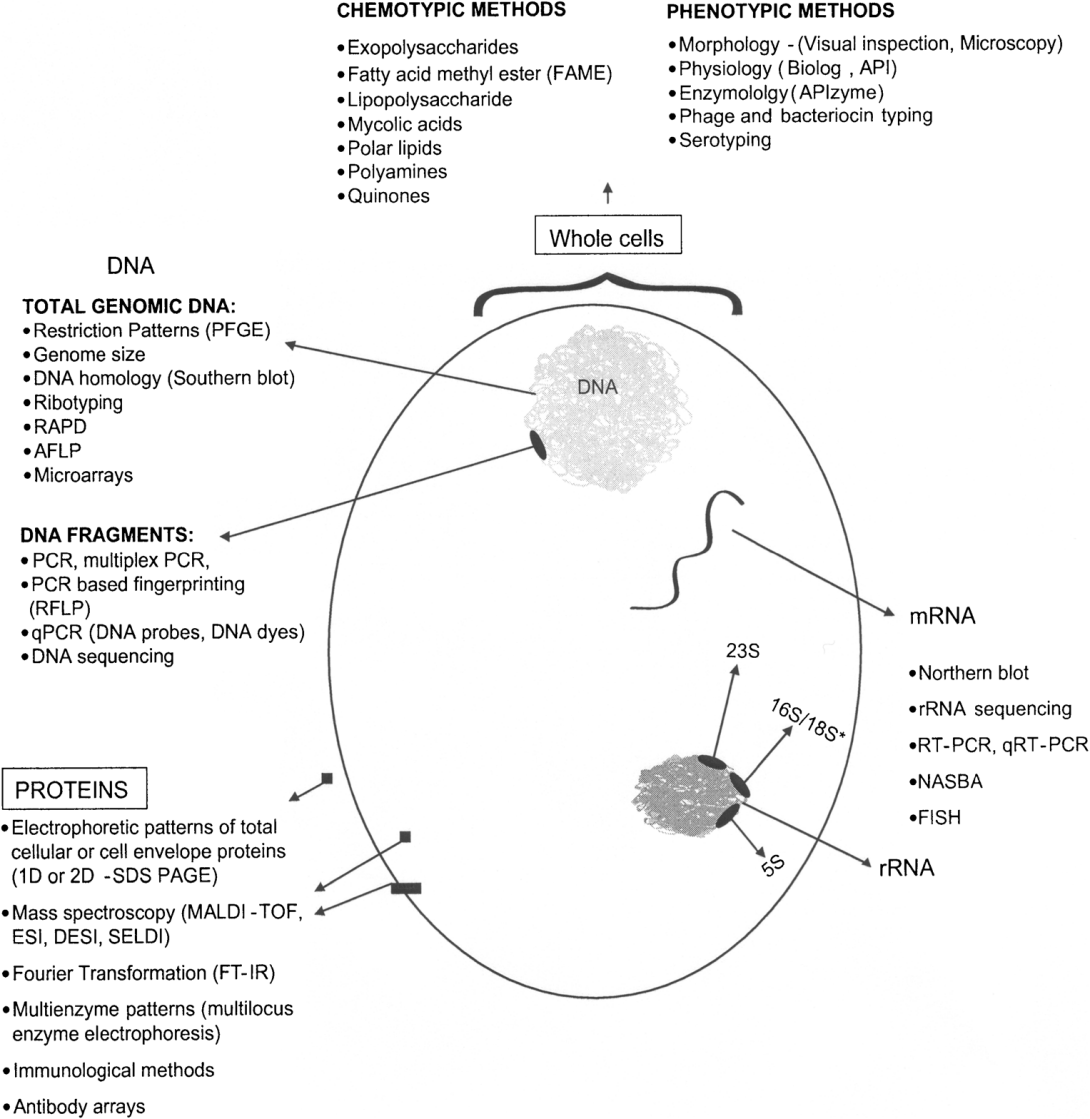
Various cell targets used for non-culture-based methods and culture-based phenotypic methods to detect microorganisms from water (from Sen and Ashbolt [56]). *PFGE* pulse field gel electrophoresis, *RAPD* random amplified polymorphic DNA, *AFLP* amplified fragment length polymorphism, *MALDI-TOF* matrix-assisted laser desorption/ionization-time of flight, *NASBA* nucleic acid sequence-based amplification, *FISH* fluorescent in situ, hybridization, *rRNA* ribosomal ribonucleic acid. *Represents RNA from 18S rRNA present in eukaryotes such as protozoa, or 16S rRNA present in bacteria

Historically, clinical and environmental microbiology methods have been based on culturing cells on selective media. Today this is still the general situation in clinical laboratories, although next-generation sequencing costs are so rapidly decreasing that single gene to whole metagenomic approaches are enabling rapid and broader detection of pathogens from clinical and environmental samples (Fig. 1). From a water perspective, qPCR assay for *Enterococcus* spp. (targeting 16S rDNA) was the first molecular method approved by the EPA (for recreational water assessment in treated/sewage-impacted water bodies [57•]). This assay of total (dead or alive) enterococci provides the best index to health risk following fresh (and marine) water exposures in epidemiology studies [58•] (gastrointestinal risk was assumed to be dominated by enteric viruses due to the presence of municipal wastewater contamination). Hence, it would seem appropriate to consider the use of qPCR for enterococci as possibly the most useful microbial index identified to date for sewage-contaminated drinking water. Furthermore, improved detection sensitivity and apparent viability appears possible by qPCR targeting the thousands of copies of 16S ribosomal RNA within viable bacterial cells rather than qPCR directed to low-copy-number DNA-based genes [59•].

As for all possible pathogen infections, we now realize that our health is also reliant upon the ‘health’ of our microbiomes and, in the case of the gastrointestinal tract, the gutome is being explored by next-generation sequencing approaches. Informed by these metagenomic studies and clinical samples from outbreak cases, the expectation is that many more important, currently uncultured drinking water pathogens (and pathogen-vulnerable gutomes) will be identified over the coming decades than those listed in Table 1, noting that etiologic agents are identified in less than 45 % of drinking-water outbreaks in the US [16•], in part due to the inability to culture them.

However, even well-known bacterial pathogens lose the ability to be cultured when moved from recent cell growth in the gut to the aquatic environment. Campylobacters are particularly well known for this, forming viable but non-culturable (VBNC) cells [60•]. Whereas the term active but non-culturable (ABNC) makes more sense given the term ‘viable’ normally refers to the ability to grow an organism, VBNC is used in this review due to the majority of papers on this topic describing these cells as such. The VBNC cell state is important to drinking water and public health due to the potential of VBNC cells to cause infection in humans [61], formed during water disinfection [62], and this has not yet been addressed by current culture-based compliance monitoring of drinking water, nor has the likely environmental biofilm niche for VBNC environmental pathogens been sampled. A further complication (in describing these pathogens as enteric and/or environmental) is that resuscitation of VBNC cells may also occur within environmental (free-living) protozoa—true for a variety of the intracellular enteric pathogens listed in Table 1 [63]. Hence, it is probably more useful to take a microbial ecology perspective and think of the VBNC state as part of the normal lifecycle of most bacteria that do not form spores. As such, various mechanisms have probably evolved over millennia, with their various interactions within aquatic (and subsequently water system) predatory eukaryotic organisms, well before they adapted to the human gut (also true for the FIB, e.g. *Enterococcus faecalis* [64]).

Of particular relevance to drinking water disinfection, and easily confused with the VBNC state, are highly-resistant cell forms that enable bacterial survival in the presence of stressors, known as persister cells [65]. Unlike VBNC cells, there is always some fraction of persisters in a population present as a reversible non-replicating state, a particular feature of AMR strains. Persisters may represent a few percent of a bacterial population and are more common post-exponential growth [65]. Although not specifically identified, persisters may contribute to the inability to completely disinfect drinking water, seen by various tailing effects in disinfection kinetic studies [66] and the downstream presence of various enteric bacteria identified by molecular methods within drinking water/biofilms, e.g. along with VBNC forms of *E. coli* and *Helicobacter pylori* [25, 67].

*H. pylori* is a particularly controversial ‘waterborne’ pathogen due to the presence of non-culturable cells in drinking water. To date, *H. pylori* has only been detected by molecular methods in drinking waters [67], and it demonstrates a subpopulation able to ‘survive’ drinking-water chlorination treatment [68], yet there is only weak epidemiologic evidence for waterborne transmission [69]. As with many enteric pathogens, person-to-person spread is probably more important than the waterborne route, yet it remains unclear if *H. pylori* should even be considered waterborne in developed regions.

### Reference Parasitic Protozoa and Fungi

When a parasitic protozoan agent is identified during waterborne outbreaks in the US it was most often due to the presence of cysts to human-infective *Giardia intestinalis* (synonyms *G. lamblia* and *G. duodenalis*) [16•], further described as assemblages A or B. However, from a European perspective, and globally in developed regions over the last decade, more disease burden has resulted from chlorine-resistant oocysts of *Cryptosporidium hominis* or *C. parvum* [70]. Considerable research and management changes have been successful in reducing waterborne cryptosporidiosis from large municipal systems, and molecular methods are now available to identify the small subset of genotypes that are likely to be human infectious [71]. Nonetheless, QMRA estimates of waterborne cryptosporidiosis from small systems in developed regions is considerably higher than giardiasis, and possibly well above levels considered acceptable [72]. Therefore, *C. parvum* and/or *C. hominis* are generally the reference parasitic protozoan used in QMRA to assess and manage drinking waters. Unfortunately, most jurisdictions do not discriminate between the genotypes that may infect humans versus those that may not (as a precautionary principle), yet that decision may have major cost ramifications, and money could be better spent elsewhere to reduce drinking-water disease burden when the two key *Cryptosporidium* oocyst sources (human sewage or calf feces [73]) are not likely to impact source drinking waters.

Waterborne outbreaks from other parasitic protozoa appear to be rarer (e.g. *Blastocystis hominis*, *Cyclospora cayetanensis*, and *Toxoplasma gondii*) [74], and attention to *Cryptosporidium* control in watersheds, water treatment, and distribution should largely address these other members. Less well-understood are the microsporidia, once classified as parasitic protozoan and identified in some waterborne outbreaks [70]. *Enterocytozoon bieneusi* has been identified in source waters [73] and is considered the most common member among 17 human pathogenic microsporidian species that largely impact HIV/AIDS and immunosuppressed patients [75]. Taxonomically, the microsporidia are within the phylum *Microspora*, and are classified among spore-forming unicellular fungal parasites. As such, they produce smaller spores than the oo/cysts of parasitic protozoa, but due to likely low occurrence their movement through sand filtration processes [76] may be better modeled using bacterial spore surrogates, which are typically removed to a lesser degree than parasitic protozoan oo/cysts [77]. Zoonotic spread from bovines is considered important, although microsporidial infection rates in cattle are probably significantly lower than for *Cryptosporidium* spp. [78].

## Water-Based (Environmental) Pathogens

### Amoeba-Resisting Bacteria

Respiratory disease caused by NTM and *L. pneumophila* is the dominant hospitalization cost claim in the US, and largely results from drinking-water-related aerosol exposures [14•]. However, there has been slow recognition of this fact, due, in part, to the overlap in clinical findings of the severe form of pneumonia known as Legionnaires’ disease with other more common causes of community-acquired pneumonia (CAP), noting that NTM also contribute to the health burden through wound and soft tissue infections, and *P. aeruginosa* via AMR infections in healthcare settings and, to a lesser degree, from folliculitis via pools and spas [79]. Looking at the legionellae first, despite *Legionella micdadei* being identified from blood in a CAP patient in 1943 it took the 1976 Legionnaires’ disease epidemic in Philadelphia to fully recognize and describe *L. pneumophila* and Legionnaires’ disease [80]. Indeed, retrospectively, the first documented epidemic of Legionnaires’ disease was traced back to Austin, MN, USA, in the summer of 1957 [81]. However, legionellosis was only made a reportable disease in the US in 2001, with *L. pneumophila* causing 80–90 % of identified cases in the US [16•] and worldwide [82], and with *L. longbeacheae* adding 2–7 % of Legionnaires’ disease cases, except in Australia where it accounted for approximately 30 % [82]. A further complication results from the difficulty in culturing *L. pneumophila*, which requires the reduced forms of the amino acid cysteine and ferrous iron [83], and the likelihood of VBNC and other difficult-to-culture cell forms [84] being dominant in drinking waters. One solution identified in the 1980s is to co-culture these problematic cell forms with free-living amoebae [85], as occurs in nature [86]. Follow-up identification can then use molecular methods targeting the 16S rRNA gene and PCR directed to the macrophage infectivity potentiator (mip) gene to confirm *L. pneumophila*, with other genes (*gyr*A, *rpo*B, *rnp*B) also used to describe additional species [87].

The NTM are ubiquitous in freshwaters and are often the dominant group within drinking-water pipe biofilms, probably selected for by the presence of a residual disinfectant [88•]. Opportunistic members linked to drinking water infections include the *Mycobacterium avium* complex (which includes *M. intracellulare*), *M. chelonae, M. fortuitum, M. gordonae,* and *M. kansasii* [89]. However, care is needed to resolve between clinically important strains and non-pathogenic environmental members. Environmental isolates of clinically relevant species are often not identified as the etiologic agents. However, is this just a limit of our culture-based approaches or truly a misdiagnosis of the environmental source? Next-generation sequencing not reliant on culturing is likely to resolve such controversies with these water-based pathogens.

Most important to note is that *L. pneumophila*, NTM, and an increasing list of other amoeba-resisting bacteria (ARB) are opportunistic pathogens growing within drinking-water biofilms (Table 1). Chronic persistence of ARB is particularly problematic to control in healthcare settings due to the ubiquitous nature of amoebae and the protection they confer on their intracellular pathogens [90]. Comparative genomics is also proving to be beneficial to identify human pathogenic members, such as key *Legionella* species [82].

### Free-Living Amoebae and Their Viruses

Both culture- and molecular-based methods have identified the free-living amoebae *Acanthamoeba*, *Naegleria*, *Protacanthamoeba* spp. and *Vermamoeba* (formally *Hartmannella*) *vermiformis*. Less frequent detections include *Echinamoeba*, *Vahlkampfia*, and *Vannella* spp., amongst others, all likely environmental hosts of amoeba-resisting bacterial pathogens such as NTM and legionellae in drinking waters [22, 91•] (Table 1). Some of these free-living amoebae may be pathogens in their own right, such as those causing *Acanthamoeba* keratitis via drinking water, a serious eye infection primarily affecting contact lens users, although several cases have involved other genera (*Vahlkampfia*, *Vannella*, and *Hartmannella* spp.) [22]. More severe and often life-threatening infections affecting immunocompetent children and immunocompromised adults include encephalitis involving *Acanthamoeba* spp. and *Balamuthia mandrillaris*, and *Naegleria fowleri* causing meningoencephalitis [22]. Nasal irrigation with drinking water from warm climatic zones has been identified as an important source of primary amoebic meningoencephalitis (PAM) caused by growth of *N. fowleri* through nasal passages to the brain [92]. Luckily, *N. fowleri* is readily controlled in drinking water by maintaining a disinfectant residual [93], remembering the main growth is thought to occur within biofilms, therefore biofilm control through minimization of stagnation zones and monitoring is also suggested for problematic climatic zones.

*Acanthamoeba polyphaga mimivirus* (APMV), first misidentified as a bacterium due to its large size (>700 nm capsid), appears to be one of a group of giant viruses that infect aquatic and soil protozoa and metazoan [94]. They are environmentally very robust and some have been implicated in human cases of pneumonia [21]. Recent viral metagenomic studies have vastly increased the number of new members of the *Megavirales* order of giant viruses and their virophages [95], with new species of clinical significance expected.

### Control of Water-Based Pathogens

A common feature of the water-based pathogens is the ability to grow to problematic concentrations within biofilms on pipe walls and sediments, particularly during periods of water stagnation and warmer conditions; therefore, control below some critical concentration is necessary to manage these environmental pathogens. For *L. pneumophila*, some millions of cells per liter of drinking water may be necessary so that aerosols of a respiratory size reach the alveoli and cause human infection [96]. Lesser concentrations, but some contact, are required for *P. aeruginosa* folliculitis [79]; the infective doses for NTM and *N. fowleri* are probably low but are undocumented.

Therefore, there are competing issues in managing all of these water-based pathogens, should they co-occur. *Legionella* and NTM of human health concern probably both grow within amoebae; therefore, limiting susceptible amoebae hosts by way of biofilm control seems logical, as would monitoring for total amoebae to evaluate control [97]. However, maintaining a high disinfectant residual may select for NTM biofilms that could contain problematic strains, although control *N. fowleri*. Some evidence suggests that monochloramine is effective against free and amoeba-cultured *L. pneumophila*, while chlorine and chlorine dioxide are less effective drinking-water disinfectants against amoeba-cultured pathogens, implying different modes of disinfection [98]. Of pipe materials, copper appears to develop less biofilm biomass, but select for VBNC *L. pneumophila* compared with PVC [99•, 100•], whereas cross-linked polyethylene appears to support both legionellae and *M. avium* complex as culturable cells at 40–55 **°**C [101]. Hence, keeping piped cold water cool (<20 **°**C) and hot water >60 **°**C via constant circulation seems to offer the most pragmatic control options for all of these pathogens within premise plumbing [15]. A speculative control approach is to maintain an actively antagonistic biofilm community that suppresses growth of members that may be opportunistic pathogens [102].

### Opportunistic Fungal Infections

Of the filamentous fungi, *Aspergillus fumigatus* and *A. terreus* have been isolated from hospital drinking waters that caused nosocomial infections [103]. However, while fungal filaments are often observed in drinking-water biofilms, they are rarely identified. Recent next-generation sequencing of drinking-water biofilms has identified three opportunistic fungal pathogens—*Candida albicans, C. parapsilosis*, and *Exophiala* (formally *Wangiella*) *dermatitidis* [104]. It is noted that disseminated candidiasis caused by *C. albicans* is a leading nosocomial bloodstream infection in the US, with a high case fatality rate. *E. dermatitidis* is a ubiquitous black yeast that grows optimally around 40 °C and is most commonly seen in saunas, steam baths, and humidifiers [105].

## Conclusions

With of advent of next-generation sequencing for routine clinical and environmental microbiology, there is renewed hope to improve upon identifying novel and currently known, but non-detected, physiological states of drinking water pathogens.

There is also intriguing new evidence that what were once thought of as strictly enteric pathogens may contain members with environmental amplification potential, ranging from human enteric viruses [34•] to *E. coli* O157:H7 [23•], but for now that is largely speculative. What is more concrete is the rising health burden resulting from opportunistic pathogens via drinking water [13], which are largely unregulated worldwide.
